# Reliability and Safety of Functional Capacity Evaluation in Patients with Whiplash Associated Disorders

**DOI:** 10.1007/s10926-012-9403-z

**Published:** 2012-11-22

**Authors:** M. A. Trippolini, M. F. Reneman, B. Jansen, P. U. Dijkstra, J. H. B. Geertzen

**Affiliations:** 1 Department of Work Rehabilitation, Rehaklinik Bellikon, Suva Care, 5454 Bellikon, Switzerland; 2 Department of Rehabilitation Medicine, Center for Rehabilitation, University Medical Center Groningen, Groningen, The Netherlands; 3 Department of Oral and Maxillofacial Surgery, University Medical Center Groningen, University of Groningen, Groningen, The Netherlands

**Keywords:** Disability evaluation, Whiplash injury, Chronic pain, Vocational rehabilitation

## Abstract

*Introduction* Whiplash-associated disorders (WAD) are a burden for both individuals and society. It is recommended to evaluate patients with WAD at risk of chronification to enhance rehabilitation and promote an early return to work. In patients with low back pain (LBP), functional capacity evaluation (FCE) contributes to clinical decisions regarding fitness-for-work. FCE should have demonstrated sufficient clinimetric properties. Reliability and safety of FCE for patients with WAD is unknown. *Methods* Thirty-two participants (11 females and 21 males; mean age 39.6 years) with WAD (Grade I or II) were included. The FCE consisted of 12 tests, including material handling, hand grip strength, repetitive arm movements, static arm activities, walking speed, and a 3 min step test. Overall the FCE duration was 60 min. The test–retest interval was 7 days. Interclass correlations (model 1) (ICCs) and limits of agreement (LoA) were calculated. Safety was assessed by a Pain Response Questionnaire, observation criteria and heart rate monitoring. *Results* ICCs ranged between 0.57 (3 min step test) and 0.96 (short two-handed carry). LoA relative to mean performance ranged between 15 % (50 m walking test) and 57 % (lifting waist to overhead). Pain reactions after WAD FCE decreased within days. Observations and heart rate measurements fell within the safety criteria. *Conclusions* The reliability of the WAD FCE was moderate in two tests, good in five tests and excellent in five tests. Safety-criteria were fulfilled. Interpretation at the patient level should be performed with care because LoA were substantial.

## Introduction

Whiplash injuries occur primarily after motor vehicle collisions, but can also occur during work, sports or other mishaps leading to an indirect cervical trauma. The Québec Task Force (QTF) on Whiplash-Associated Disorders (WAD) defined whiplash as “an acceleration-deceleration mechanism of energy transferred to the neck that results in soft tissue injury that may lead to a variety of clinical manifestations including neck pain and its associated symptoms” [1]. Patients with WAD may also suffer from upper limb pain, paresthesias, psychological distress, anxiety, dizziness, headache, fatigue, nausea, concentration deficits and many more symptoms [2, 3]. WAD refers to the clinical entities related to the injury, but should be distinguished from the injury mechanism [1].

Whiplash injury incurs large economic, social and personal burden. Recent studies report that 10–40 % of patients with WAD will fail to recover [1, 4, 5]. If recovery occurs, this will take place within the first 2–3 months [6]. The WAD Task Force proposed that patients with WAD who do not return to work within 6–12 weeks after injury receive an interdisciplinary assessment including disability measures so that interventions may be specifically directed, potentially averting the course to chronicity [7, 8].

Functional capacity evaluations (FCE) were developed to assess work-related abilities [9, 10]. These work-related tests were based on a taxonomy described in the US Department of Labor’s Dictionary of Occupational Titles (DOT) [11]. Although no consensus on the definition of FCE is available [12], we use the term as follows: FCE is an evaluation of the capacity to perform activities that is used to make recommendations for participation in work while considering the individuals’s body functions and structures, environmental factors, personal factors and health status. During the past decade, measurement properties of FCEs such as reliability, validity and safety have been determined [13]. However, these measurement properties have mainly been investigated in patients with LBP [14] and, to a lesser extent, in healthy subjects [15] and patients with the early stages of osteoarthritis of knees and hips [16], work-related upper limb disorders [17], and work-related neck disorders [18]. Moreover, there is a lack of knowledge on measurement error of FCE, which seriously limits clinical decision making. Furthermore it has been proposed to perform FCE in a more specific and efficient way by selecting a limited number of activities targeted to the workers condition [19, 20]. To date no specific FCE for WAD has been developed. The safety of work-related assessments has been recognized as a necessary attribute of FCE studies [21, 22], but safety issues such as pain-reaction, muscle soreness, adverse effects and pain medication use have not been reported in patients with WAD.

Hence the aim of this study was to analyze test–retest reliability, measurement error and safety of FCE in patients with WAD who did not return to work within 6–12 weeks after injury and who received workers’ compensation.

## Methods

### Participants

Participants from all over the country (Switzerland) were referred by either a physician or a case manager of the workers’ compensation insurance for a half-day comprehensive interdisciplinary rehabilitation assessment. Participants were referred when they had not regained full working capacity within 6–12 weeks after initial injury. From January to October 2011 from, n = 71 patients, with WAD were asked to participate in this reliability study after they had completed their FCE. Inclusion criteria were if participants had symptoms according the Québec Task Force-Classification of WAD, grade I (pain, stiffness or tenderness without physical signs) or grade II (pain, stiffness, or tenderness with decreased range of motion and point tenderness), main pain in the head or neck region, sufficient German language skills to communicate with the assessors (all questionnaires were available in German and five foreign languages spoken by the participants), an age of 18–65 years, and willingness to participate (signed informed consent). Exclusion criterion was co-morbidity which considerably limited function, such as neurological deficits, rheumatoid diseases, spinal fractures, tumors, osteoporosis, psychiatric disorders, pregnancy, cardiac hypertension etc. Based on convenience, a sample of participant was selected by an independent person, not involved in the testing procedure, to participate in the retest.

A convenience sample of 4 physiotherapists (2 female, 2 male) conducted the FCEs. All attended the official 2 day FCE training course, are accredited as FCE-Therapists by the Swiss Association of Rehabilitation [23], had performed at least 20 one day FCEs in the previous year (median 28, interquartile range (IQR), 21–37) and had a minimum of 2 years working experience in vocational rehabilitation (median 7 years, IQR 2–14), and a minimum professional practice experience of 2 years (median 14 years, IQR 4–23). For this study, all physiotherapists received an additional half day training, and had a FCE supervised by an FCE expert.

### Procedure

All participants received written and verbal information about the study. Participants were informed that they would be allowed to withdraw their participation at any time without disclosing reasons and without consequences for their medical care. The Medical Ethics Committee of Canton Aargau granted the ethical approval for this study (EK AG 2010/055). Participants received reimbursement of travel expenses and 50 Swiss francs after completion of the second FCE session.

#### Study Design

A test–retest design was used. During the first visit a review of the medical history and a physical examination was performed by a physician lasting approximately 60 min, followed by FCE administered by a physiotherapist. Administration of the WAD FCE lasted approximately 60 min.

After the first FCE participants were asked whether he would want to participate in a retest. The fixed order of the tests was standardized and constant between sessions. The second FCE was conducted 1 week later (median 7 days, IQR 6–7). This time period between the two tests needed be long enough to reduce carry-over effects and delayed muscle soreness [24], and short enough considering that the health condition of the study participants may still change. The second FCE was administered by the same tester. Time and day for the retest session were held constant as much as possible. Participants and testers were blinded to the results of the first FCE.

### Measures

#### Functional Capacity Evaluation

The FCE applied in this study (WAD FCE) consisted of 12 tests, based in part on the WorkWell FCE (formerly the Isernhagen Work System) [25]: handgrip strength (left and right), lifting floor to waist, lifting waist to overhead, short two-handed carry, long right- and left-handed carry, overhead work, repetitive reaching (left to right and right to left [17], 50 m walking test [26] and a 3 min step test [27]. Test descriptions are presented in the Appendix. Participants were briefly instructed on how to perform each test. The evaluator first gave a single demonstration of each test. Participants were then asked to perform the tests to their maximum ability. Weights lifted were gradually increased according to a participant’s performance, using weights of 2.5 and 5 kg. To determine the physical effort level, testers used observational critera [23, 25]. Testing could be terminated for four reasons: the participant stopped because of, for example, pain; the observer deemed testing to have become unsafe based on biomechanical criteria; heart rate exceeded 85 % of the age-related maximum (220 minus age of participant); or a predefined time limit was reached.

#### Safety

Safety of the FCE was assessed by heart rate monitoring, observational criteria for effort level during work related tasks, pain reaction as measured with the Pain Response Questionnaire (PRQ) [24], additional pain medication, or reports of serious adverse effects. Participants were asked to score their pain for 17 separate body regions in an 11-point NRS, in which 0 was “no pain” and 10 was “worst pain”. Participants were also asked whether their pain was attributable to muscle soreness, to a different origin, a combination of these, or of unknown origin. The participants were asked to fill in the PRQ on the subsequent days (using a diary) after the first WAD FCE until the day of the retest. The WAD FCE was considered safe under the following conditions: when the heart rate did not exceed the age-related maximum, when it did not exceed the maximum observational criteria for effort level during work-related tasks, when it did not lead to injuries, when it resulted in no serious adverse effects, when it did not increase by more than three NRS points [28], and when reported muscle soreness increased in the first 24–48 h (which is a normal response), subsided during the following 2 days and then returned to pretest levels within 5–7 days [24]. A response which did not adhere to this definition was interpreted as an abnormal response.

#### Additional Measures

##### Participants Characteristics

Participant characteristics included age, gender, marital status, education, nationality, work status, current litigation, and compensation-status, among others. Pain intensity was measured with an 11-point numeric rating scale (NRS) [29].

##### Disability

Neck pain-related disability was measured with the Neck Disability Index (NDI) [30]. The NDI contains 10 items, ranging from no disability (0) to total disability (5). The maximal overall score is 50 points (complete disability).

##### Anxiety and Depression

Anxiety and depression were measured using the Hospital Anxiety and Depression Scale (HADS) [31]. The HADS consists of two scales, one for anxiety and one for depression. Each scale contains seven items, with each item rated from 0 (best) to 3 (worst). The scale scores are calculated by summing the responses to the items up to a maximum score of 21 points per scale (severe case) [32].

##### Self-efficacy

Self-efficacy in execution of activities which involve the spine was measured with the Spinal Function Sort (SFS) [33]. The instrument contains 50 drawings with simple descriptions of activities that involve the spine. Participants rated self-efficacy for each activity from “able” (4) to “unable” (0). The SFS yields a single rating ranging from 0 to 200.

### Data Analysis

Depending on data-distribution, test and retest data were analyzed using parametric or non-parametric statistics. Test–retest reliability was expressed as an Interclass Correlation Coefficient (model 1; one-way random) (ICC). ICC was interpreted as follows: ICC ≥ 0.90 is excellent; good when ICC was between 0.75 and 0.90; moderate when ICC was between 0.50 and 0.75; and poor when ICC ≤ 0.50. ICCs were acceptable when ICC ≥ 0.75, and the lower boundary of the 95 % confidence interval of the ICC ≥ 0.50 [34]. Agreement was expressed in limits of agreement (LoA) (mean difference ± 1.96 × standard deviation of mean difference) [35]. The ratio between the limits of agreement and the mean score of two sessions was calculated (LoA/mean of two sessions) × 100 %), to determine the relative width of the LoA, and to allow comparison of LoA to other studies. Paired t-tests were used to analyze systematic differences between the first and second test session. A response which did not accord to this definition was interpreted as an abnormal response. An analysis was performed to identify differences between those participants who completed two sessions and those who only completed one session. All analyses were performed with SPSS (Statistical Package for Social Sciences, Version 19).

## Results

Of the eligible participants, 32 (45 %) completed both sessions, and 39 (55 %) did not participate in the retest. The reasons for not participating were as follows: 21 (54 %) of participants were working at the time of the retest; 6 (15 %) explicitly did not want to participate with no reason declared; 4 (10 %), did not feel capable due to temporary pain increase at the time of the first WAD FCE; and 8 (21 %) mentioned other reasons, such as being on holiday, no transport facilities available etc. A total of 32 participants performed all of the tests. Demographic and clinical variables of the study sample are presented in Table 1. The four physiotherapists conducted between 6 and 11 WAD FCEs each.

**Table 1:** Participants characteristics (n = 32)

	Mean (SD)
Age (years)	39.6 (12.3)
BMI	28.2 (5.4)
Disability (NDI 0-50)	21.7 (5.8)
Anxiety (HADS 0-21)	7.3 (4.3)
Depression (HADS 0-21)	6.1 (3.6)
Self efficacy (SFS 0-200)	146.4 (31.6)
Injury duration since (days), SD	89.6 (33.9)
	n or %
Work capacity for the own job (in %) at the time of WAD FCE^a^	62.8 % (38.5)
Gender: female	11 (34 %)
Marital status: married	9 (28 %)
Nationality: Swiss	22 (69 %)
Education low^b^	10
Intermediate	21
High	1
Physical work demands^c^ (kg)	n
0–10	10
11–25	8
25–50	9
>50	5

^a^Work capacity was assessed by the referring physician

^b^Low = no vocational education, intermediate = vocational education, high = bachelor or higher education

^c^ Physical work demands according to the Dictionary of Occupational Titles (DOT)

*BMI* Body mass index formula: weight (kg)/height (cm)^2^, *NDI* Neck Disability Index, *HADS* Hospital Anxiety and Depression Scale, *SFS* Spinal Function Sort

### Reliability and Agreement

ICC ranged between 0.57 and 0.96 (Table 2). Ratios of the LoA of the WAD FCE tests were between 15 % (50 m walking test) and 57 % (lifting waist to overhead). Bland and Altman plots revealed variances that were not related to the magnitude of the outcome (plots not shown). The mean performance of the participants increased in the second session in 8 WAD FCE tests, of which three were statistically significant results (*p* < 0.05).

**Table 2:** Test results of 2 WAD FCE sessions, and limits of agreement and intra class correlation between the test results

WAD FCE items	Mean session 1	SD session 1	Mean session 2	SD session 2	Mean difference	SD of mean difference	95 % CI of mean difference	*p*	LoA	Ratio of LoA (%)	ICC	95 % CI of ICC	Inter-pretation of ICC
Hand grip strength right (kgF)	37.8	14.6	40.5	14.3	−2.6	5.3	−4.5 to −0.7	.008	−13.0–7.7	26	0.92	0.84–0.96	Excellent
Hand grip strength left (kgF)	35.0	14.7	38.7	13.6	−3.6	5.8	−5.8 to −1.5	.001	−15.1–7.8	31	0.89	0.78–0.94	Good
Lifting floor to waist (kg)	24.1	9.7	24.7	8.9	−0.6	3.8	−2.0 to 0.7	.354	−8.0–6.7	30	0.92	0.84–0.96	Excellent
Lifting waist to overhead (kg)	13.8	5.8	15.3	4.9	−1.5	4.2	−3.0 to 0.0	.054	−9.8–6.8	57	0.66 0.80^a^	0.42–0.82 0.64–0.90^a^	Moderate Good^a^
Short carry two handed (kg)	32.5	12.7	33.2	13.2	−0.7	3.6	−2.0 to 0.6	.288	−7.7–6.4	21	0.96	0.92–0.98	Excellent
Long carry right handed (kg)	20.9	6.9	20.2	6.5	0.6	3.0	−0.5 to 1.7	.255	−5.3–6.6	29	0.87	0.90–0.95	Good
Long carry left handed (kg)	19.4	6.3	19.5	5.7	−0.1	2.6	−1.1 to 0.8	.759	−5.2–4.9	26	0.91	0.83–0.96	Excellent
Overhead working (sec)	223.0	97.3	227.7	90.9	−4.7	55.8	−24.8 to 15.4	.636	−114.1–104.7	49	0.83	0.68–0.91	Good
Repetitive reaching right (sec)	77.2	24.5	72.0	21.2	5.3	13.6	0.3 to 10.2	.037	−21.5–32.0	36	0.81	0.64–0.90	Good
Repetitive reaching left (sec)	77.1	24.5	72.6	22.0	4.5	14.0	−0.5 to 9.5	.078	−22.9–31.9	37	0.81	0.65–0.90	Good
50 m walking test (km/h)	5.1	0.9	5.2	1.0	−0.1	0.4	−0.2 to 0.1	.362	−0.9–0.7	15	0.91	0.82–0.95	Excellent
3 min step test (mean heart rate after 1st min)	116.8	29.7	116.8	20.7	9.0	24.0	−8.7 to 8.9	.988	−46.9–47.0	40	0.57	0.28–0.77	Moderate

^a^Results of an analysis when 1 participant who refused to lift any weight in the first session, was excluded from the analysis (see discussion)

*SD* standard deviation, *LoA* limits of agreement, 95 % CI 95 % confidence interval, *ICC* interclass correlation coefficient

Ratio of LoA (%): the ratio between the limits of agreement and the mean score. [(1.96 × standard deviation of mean difference)/mean session 1 and 2 × 100 %]

### Safety

**Fig. 1:**
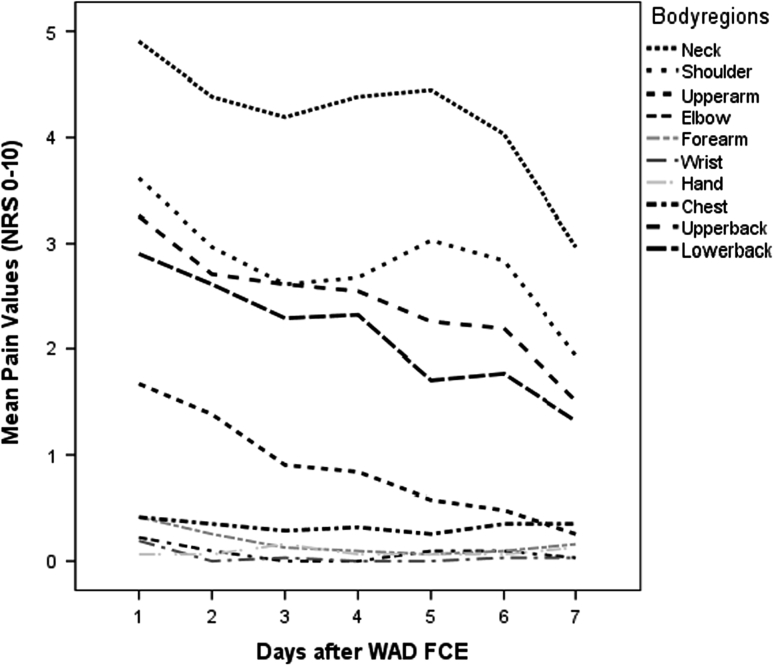
Means of the reported pain response per day after WAD FCE measured by the pain response questionnaire (PRQ)

Except for one participant who had to stop the material handling test because his/her heart rate reached in excess of 85 % maximum, all the WAD FCE tests were completed before the 85 % maximum heart rate was reached. At the endpoint of each of the material handling tests of the first test session, the mean heart rate difference to the theoretical age-related maximum was 35.9 (SD 16.6). The mean NRS pain before the first WAD FCE was 4.3 (1.8), and 5.3 (SD 1.9) after WAD FCE, *p* value < 0.001 (mean change −1.1, SD change 1.3). For the second WAD FCE session, these values were 4.3 (SD 1.9) for NRS pain before and 4.9 (SD 1.8) for NRS pain after, *p* value < 0.001 (mean change 0.6, SD change 1.1). On an individual level, pain increased by two or more NRS points in 18 participants (57 %), with none exceeding three points. Symptoms also decreased to a mean at pre-test levels in 7 days. Average pain scores in the neck and shoulder region measured with the PRQ decreased after the second day post WAD FCE (Fig. 1). One participant did not complete the PRQ and was excluded. No serious adverse events were reported during or after test and retest.

### Differences Between Participants and Non-participants

On average non-participants performed less than participants. We performed a Mann–Whitney U Test for independent-samples to compare the WAD FCE results of the first session between the group that was retested and the group that was not retested (non-participants). In nine out of 12 WAD FCE tests, the results showed no significant difference between the groups. In the three WAD FCE tests with the significantly different test results, the non-participants lifted less in a short two handed carry test (Mean 24.4 kg, SD 12.7), and in the long carry one handed test (Mean right: 16.9 kg SD 7.7; Mean left: 16.3 kg, SD 7.4). Additionally, we compared clinical characteristics, such as neck pain disability, anxiety, depression levels, self-efficacy and pain scores. These characteristics did not differ significantly between participants and the non-participants.

## Discussion

### Reliability

The test–retest reliability out of 11–12 WAD FCE test items was good to excellent. Healthy volunteers [15], patients with chronic LBP [14] or patients with osteoarthritis of hip and/or knee [16] showed smaller variability in this FCE test compared to the WAD FCE. The following reasons may explain these differing results. In the case of healthy volunteers, who are less affected by pain, less variability in the test results is expected. FCE in the capacity of a patient with chronic low back will not change between two sessions because they are in a relatively stable i.e. chronic phase of the illness. The study of osteoarthritis patients [16] involved conducting the retest study 1 day after the first test session, therefore a lower variability may be explained by recall bias due to the limited time between the two test sessions. As expected from WAD patients suffering from pain in the neck region, larger LoA scores were observed in the tests affecting the upper body regions i.e. “overhead work” and “lifting waist to overhead”.

Lifting from waist to overhead had a moderate ICC (0.66), with significantly different values recorded between the first and second session. This result was in part due to a participant who refused to lift any weight overhead in the first session, but lifted 15 kg in the second session. An post hoc sensitivity analysis was performed by excluding that participant from the analysis. The ICC value then increased to 0.80, which indicated good reliability.

Regarding the overhead work test with an ICC of 0.83, the larger LoA ratios may also be partly explained by the longer duration of the test at 5 min, compared to the maximum of 90 s in the material handling tests. The longer a test, the greater the chance that the patient would perform differently in another test session. For example, in the study of Brouwer et al. [14], the reliability expressed as an ICC of a 15 min overhead work test was 0.36. To prevent ceiling effects, other researchers have modified the overhead work test by having the patients wear two cuff-weights of 1 kg around their forearm [36]. This procedure results in a reduction of endurance in the overhead work in healthy participants, and an ICC of 0.90 [17]. The results of the hand grip force (in position 2 of the Jamar hand dynamometer) proved to have good to excellent reliability, similarly to the findings of previous studies on hand grip force [37], underlining its clinical use in the evaluation of grip strength in several musculoskeletal disorders. In the repetitive reaching test, ICC values were slightly higher in WAD patients when compared to healthy participants, while LoA were between −21.5 and 32.0 in WAD patients and −9.0–12.6 in healthy participants [17]. Tests results of the 3 min step test and 50 m walking test did not change significantly between the two sessions compared to the materials handling tests. It is very unlikely that endurance and gait speed would improve in that length of time between the two sessions. Our participants were a sample of patients with sub-acute WAD, whose health status was still subject to possible change (improvement). The time interval between the two sessions therefore had to be far enough apart to avoid fatigue, learning or memory effects, but not too far apart to allow a change in health status. We therefore chose a time interval of 7 days to take these factors into account. This time period was shorter than previous reliability studies, which had time intervals of 10–21 days [14, 17, 38]. Clinically the measurement error of the test under investigation lies within ±95 % LoA. This means that, at the individual level, a patient’s performance could be considered to be changed when it exceeded the LoA. For example in “lifting floor to waist”, a patient’s performance improved if his performance increased by more than 6.7 kg.

Large limits of agreement scores in health outcome measure are common in pain patients [33, 39, 40]. As already stated there are no cut-off points of LoA [41]. However one study from Keller et al. [42]. who calculated the LoA for the Astrand bicycle test and other back strength tests in LBP patients judged a test with LoA of ≥42 % as unreliable. Based on this arbitrary cut-off value, 2 out of the 12 tests of the WAD FCE would be classified as unreliable. This large within-patient variance may be attributed to measurement and random errors of test procedure, evaluator inconsistencies, and patient behavior being influenced by motivation or pain. As hypothesized by others [14, 43], but not tested in this study, we argue that a large part of the variance can be attributed to variation within the patients.

### Safety

In a Delphi Survey of FCE experts, safety was defined as: “a situation that, given the known characteristics of the person, the procedure should not be expected to lead to injury” [12]. We controlled for safety by using self-report measures such as the NRS, with a diary questionnaire, the PRQ, and measurements taken by the physiotherapist (e.g. heart rate, observation criteria). Based on our results of the PRQ, as reported in Fig. 1, we conclude that the WAD FCE temporarily increased pain at a similar rate to healthy volunteers [24] and patients with LBP following FCE [21]. Similarly to both other studies, symptoms in WAD patients also decreased within a week. No safety problems were encountered, and heart rate increased only moderately, with only one patient reaching the 85 % heart rate limit in the WAD FCE tests. From the eligible 71 patients, 4 refused to participate due to temporary pain increase directly after the first FCE session. None of these, nor any other participant, reported a formal complaint and no serious adverse effects were reported. We therefore believe that safety was not compromised.

### Limitations and Strengths of the Study

A limitation of this study was that only 45 % of the eligible 71 participants were willing to participate in the second session. The main reason was: lack of time (most were already returned to work, others were on holiday, or were living a long distance away etc.). The same phenomenon was found in a FCE test–retest study of Brouwer et al. [14] were approximately 100 patients were eligible during 1 year, but only 30 patients were willing to participate. In most instances, reasons for not participating were that testing would take too much time, which is similar to the Brouwer et al. study. It is unknown how non-participants would have influenced reliability of the WAD FCE tests. As learning effects influence test–retest reliability [44, 45], we did not inform participants of the detailed test results, and ensured the memory effect was minimized by maintaining a large enough time interval between test occasions. Additionally, all test protocols from the first session were collected immediately after the test procedure by an independent person, who was not involved in the testing procedure. Test protocols remained inaccessible for the testers involved. Results of paired t-tests between the two test occasions showed a general trend towards a slightly increased performance on the second occasion. This is in line with test results of healthy volunteers, who scored on average higher on the second test session [15, 17]. Although we did not expect test effects such as increased strength and mobility after the first testing session, other effects, such as increased self-efficacy, reassurance etc., may have occurred, creating consistent change within participants. Such a systematic effect will not necessarily affect reliability coefficients [44].

In our study 30 % of non-native Swiss patients participated in the study, which is a slight overrepresentation compared to the general Swiss population with 23 % with non-native citizens [46]. This is in contrast to previous FCE reliability studies [14, 16, 38] where mainly native citizens participated. Results of interventions may vary considerably between native and non-native patients [47], but to our knowledge, this has never been the subject of a study in a setting similar to ours (performance testing, reliability, agreement, safety). We therefore think that the results, although taken from a small study sample, might support the utility of the WAD FCE in non-native patients.

Secondly our testers were selected from a sample of 24 physiotherapists. The range of clinical experience covered a wide range of experience (from very low to extensive) encountered in clinical daily practice. Contrary to previous reliability studies where very experienced clinicians performed the FCE tests [6, 16, 37], our sample of assessors covered a wider range of working experience and age. This might strengthen the generalizations of the results of this study. Our study was conducted in a “real world” environment where patients with delayed recovery were sent to the WAD FCE, compared to some previous FCE reliability studies based on video analysis [43, 48].

Participants were referred by physicians and case managers from the German speaking part of Switzerland; to what extent this referral resulted in a population different from other WAD populations is unknown. Because the clinical characteristics of the non-participants did not differ from the participants, nor did the majority of test results, we assume that the selection procedure did not introduce bias relevant for the outcomes of this study (i.e. reliability, agreement, safety). Since the majority of WAD patients are suffering from WAD Grade 1 and 2 [49], the results of this study may be applied to patients with WAD Grade 1 and 2 who are still suffering from WAD 9–12 weeks after injury and are not working due to WAD.

## Conclusion

In conclusion, we observed a good to excellent test-reliability in the majority of the WAD FCE tests, while safety-criteria were fulfilled. Clinical interpretation at the individual patient level should be performed with care, however, because of the large LoA.

## Appendix: Materials and Procedures of the WAD FCE

### Isometric Hand Grip Strength

Isometric hand grip strength was measured in a seated position. The subjects held their shoulder adducted without internal or external rotation, elbow flexed at approximately 90° and the forearm and wrist in neutral position. Grip strength of the right and left hand was measured in a three-trial procedure while maintaining in a hand dynamometer in a one handgrip position (Jamar PC 5030, Preston Corporation, 1994). An average amount of kgF was scored.

### Material Handling Tests

All lifting tests were executed with a wooden crate (40 × 30 × 26 cm) of 2.5 kg, and four to five weight increments of 2.5 kg or 5 kg each were used until the maximum amount of weight was reached. Maximum performance was recorded in kg.

Lifting floor to waist was measured after five lifts of the crate from floor to table and vice versa (time limit < 90 s): hands remained on the crate during the test.

Lifting waist to overhead was measured during lifting of the crate from table to crown in standing position, and vice versa.

Two-handed carrying of a crate for a short distance was measured after five carries of 1.5 m distance at waist height. Hands remained on the crate during the test.

The one-handed carrying of a wooden crate for 15 m within 90 s began with the right hand and thereafter the left hand.

### Overhead Work Test

Overhead working was performed standing with hands at crown height for manipulation of nuts and bolts. The time that the position was held was recorded (sec).

### Repetitive Reaching Test

Repetitive reaching was determined by fast horizontal movements of the upper extremity in a sitting position. Marbles were removed from bowls at arm length distance at table height from left to right and vice versa, with right and then left arm. The time taken to remove 30 marbles was recorded (sec) [17].

### 50 m Walking Test

The walking test was executed on a 50 m-distance track. Participants were asked to walk as fast as possible. The instruction was: “Pause is allowed. Do not run!” The time taken to walk for 50 m was measured (sec), and km/h was calculated [26].

### 3 Minute Step Test

For the 3 min step test, the participant was asked to step at a constant step rate of 96 per min for a duration of 3 min. The height of the step was 30 cm. Heart rate was measured in a seated position directly after the end of the test at 30 s, and then at 60 s. The three measurements were averaged, and compared to reference data [27].
